# A qualitative study exploring perceived barriers to infant feeding and caregiving among adolescent girls and young women in rural Bangladesh

**DOI:** 10.1186/s12889-015-2115-5

**Published:** 2015-08-11

**Authors:** Kristy M. Hackett, Umme S. Mukta, Chowdhury S. B. Jalal, Daniel W. Sellen

**Affiliations:** Dalla Lana School of Public Health, University of Toronto, 155 College St, Toronto, Ontario M5T 3M7 Canada; Research and Evaluation Division, BRAC, Dhaka, Bangladesh; Micronutrient Initiative, Ottawa, Canada

## Abstract

**Background:**

Infant feeding and caregiving by adolescent girls and young women in rural Bangladesh remains relatively understudied despite high potential vulnerability of younger mothers and their children due to poverty and high rates of early marriage and childbearing. This key knowledge gap may hamper the effectiveness of maternal, infant and child health interventions not specifically tailored to teenage mothers. This study aimed to narrow this gap by documenting key barriers to optimal infant and young child feeding and caregiving perceived by adolescent girls and young women in rural Bangladesh.

**Methods:**

Focus group discussions and in-depth semi-structured interviews were conducted with 70 adolescent girls and young women participating in a community-based adolescent empowerment program in two rural regions of northwestern Bangladesh. Participants were stratified into three groups: unmarried, married without child, and married with child(ren). Thematic analysis was performed to elucidate dominant ideas regarding challenges with child feeding and caregiving across participant strata.

**Results:**

Participants in all three strata and in both geographical regions attributed actual and anticipated caregiving difficulties to five major contextual factors: early marriage, maternal time allocation conflicts, rural life, short birth intervals, and poverty. Indications are that many girls and young women anticipate difficulties in feeding and caring for their future children from an early age, and often prior to motherhood. Participants articulated both perceived need and unmet demand for additional education in infant and young child feeding, childcare, and family planning techniques.

**Conclusions:**

Provision during adolescence of appropriate education, services and financial aid to support best practices for infant feeding and childcare could significantly improve maternal self-efficacy, mental health, nutrition security and young childcare, nutrition and health in rural Bangladesh. Lessons learned can be applied in future programs aimed at supporting adolescent women along a continuum of care.

## Background

In recent years, interest in adolescent girl-child development interventions has increased worldwide, with foci ranging from health and education to economic empowerment [1–3]. This focus emerged in response to calls by the United Nations (UN) and other international bodies for increased investment in young people and emphasis on adolescence as a development window of opportunity offering potential demographic "dividends" [2, 4]. According to the UN, adolescence (the period between 10–19 years of age) is a critical life stage for all forms of human development because when poor adolescent girls become mothers to impoverished children, poverty and inequities are passed on to the next generation [2]. In low-income settings, adolescents are less likely to finish school and are more likely to be married early, leading to early childbearing and increased risk for maternal mortality and morbidity. Globally, child marriage (under 18 years of age) affects more than 10 million girls annually with nearly half of these marriages occurring in South Asia [5].

Despite national laws forbidding child marriage, marrying of young girls once puberty commences is commonplace in rural Bangladesh. According to national surveys, 64 % of Bangladeshi women aged 20–24 were married [6] and 46 % became mothers before the age of 18 [7], a large majority within marriages. Girls are often considered an economic burden on families, and arranged marriages during early adolescence are thought to relieve financial stress on natal families. A perception that girls are costly to families may be linked to the practice of dowry (goods or monies transferred from the bride’s to the groom’s family) and create incentive for early arranged marriages because dowry prices tend to increase as girls age [4, 8]. Early marriage in Bangladesh is associated with higher rates of intimate partner violence [9], high education dropout rates and decreased earning potential [4].

Teenage pregnancy is linked to multiple negative health outcomes for both mothers and infants. A study in Bangladesh found that pregnancy and lactation during adolescence halted linear growth, and led to weight loss and depletion of fat and lean body mass of young girls [10]. In addition, early pregnancy is associated with a higher incidence of preterm births and low birth weight, and thus poses significant threats to infant morbidity and mortality [11]. Despite these risks, research has shown that improved caregiving, including infant and young child feeding (IYCF) practices can be effective in reducing the negative effects of poverty and low maternal education on child nutritional outcomes [12].

Sub-optimal IYCF practices such as non-exclusive breastfeeding, delayed initiation and/or early cessation of breastfeeding, and inappropriate complementary feeding contribute to high rates of undernutrition and mortality among children in developing countries [13]. Research in Bangladesh and elsewhere has documented the protective effects of exclusive breastfeeding and age-appropriate complementary feeding on child growth outcomes [14–16]. IYCF practices are determined by a complex interplay of contextual factors including maternal education, socioeconomic status, community beliefs and feeding preferences, exposure to media and healthcare workers, and low knowledge of optimal feeding behaviours [17–21]. Behaviour change interventions are commonly employed to improve caregivers’ awareness of healthy IYCF practices, however simple delivery of education is insufficient. To ensure healthy feeding behaviours, women and their families must have access not only to relevant information, but also to appropriate support systems that address the various physical, social, and logistical determinants of infant feeding [22]. Provision of both information and practical support may boost a woman’s confidence in her ability to actually carry out healthy IYCF practices.

While a great deal of research exists on the social context of IYCF and childcare among adult women and mothers globally, less is known about the experiences of adolescent girls and young women, specifically. This underrepresentation is surprising, given the high rates of early marriage and premature childbearing in countries like Bangladesh. In order for any adolescent-targeted development programs to be effective, it is necessary to understand the social and cognitive worlds of its intended beneficiaries. The aim of this research was to a) assess adolescent perception and knowledge of key international IYCF recommendations (published elsewhere) [23]; and b) document key barriers to optimal IYCF and caregiving perceived by adolescent girls and young women in rural Bangladesh (the focus of this paper). To our knowledge, this study is among the first in Bangladesh aimed at understanding the perceived barriers to feeding and care not only among young mothers but also among girls who have never given birth. Results reported here have already informed the design of educational modules for one existing adolescent empowerment program in rural Bangladesh, and may have broader value.

## Methods

This was a descriptive qualitative study involving the collection of cross-sectional data through focus group discussions (FGDs) and open-ended semi-structured interviews (SSIs). Data was collected in April 2010.

### Programmatic context

This study was conducted within the context of *Social and Financial Empowerment of Adolescents* (SoFEA), a community-based program run by BRAC^1^. The SoFEA program targets girls aged 11–21 years throughout the country, providing livelihoods training, as well as social and financial support activities delivered through village-level clubhouses available only to female club members. Details of SoFEA program aims and activities are elaborated elsewhere [23]. At the time of this study, the SoFEA program curriculum did not include any nutrition or childcare-focused education modules or activities, however management and staff members were eager to include these components in the program. This research was therefore timely as it helped to identify specific IYCF and childcare issues most relevant to adolescent club members.

### Study setting & participants

Data were collected in Rajshahi and Pabna, two districts in Rajshahi Division, a largely rural area (82 % of households) located in the northwestern corner of Bangladesh [24]. Both districts are similarly situated in relation to the capital city (197 km and 122 km from Dhaka, respectively), and SoFEA program coverage was similar in both areas. At the time of data collection, the average age at first marriage in Rajshahi Division was 15.2 years for women, and 22. 4 years for men. Teenage pregnancy and motherhood is common in this region; in 2011 about 33 % of women between the ages of 15–19 years had begun childbearing [24].

### Participant eligibility

Young women were eligible to participate in the study if they were SoFEA club members and at least 15 years of age. We excluded from recruitment SoFEA club members younger than 15 years because this would have required parental assent. Most older club members were married at a young age and began childbearing as teenagers, and we anticipated they would have valuable and programmatically relevant perspectives to share that would also facilitate interpretation of, and comparison with, responses offered by adolescents. Therefore, while the definition of adolescence is typically between 10–19 years of age [2], SoFEA club members older than 19 years were eligible to participate to ensure equitable opportunity for study participation among all young women already enrolled in the SoFEA program.

### Sampling and participant recruitment

Participants were recruited through the SoFEA program’s enrolment records, using a stratified purposeful sampling technique [25]. We visited three SoFEA clubs in each of the two districts, which were purposefully selected to ensure that enough young women from each reproductive stratum of interest could be enrolled. Club selection was informed by the advice and expertise of the SoFEA program area managers from each district. Enrolment records were used to determine which clubs had the highest proportions of members in each of the three strata. We reasoned that young women would be more comfortable to speak openly among peers in the same reproductive stage; therefore FGDs were composed of participants from the same stratum of interest, rather than mixed. This design was also chosen to facilitate comparison during analyses.

Potential participants were initially approached by SoFEA program area managers and training staff in Rajshahi and Pabna, who arranged for researchers to meet with 10–12 eligible participants in each clubhouse, ensuring that between 6–8 of them fell into the specified marital and reproductive stratum of interest. The second author (UM), who is fluent in Bangla, invited these individuals to participate in a FGD. The remaining SoFEA club members were invited to participate in one-on-one SSIs led by the first author (KH).

### Data collection

FGDs and SSIs were utilized as a means of methodological triangulation [26]. To explore differences at various life stages we stratified the data collection and subsequent analysis by three groups based on the following participant characteristics: 1) unmarried; 2) married without child(ren); and 3) married with child(ren). Following the focus group methodology described by Miles and Huberman [27], we conducted one FGD within each stratum of participants, in each of the two districts, for a total of six discussions. We also conducted three SSIs with a participant from each stratum, in each district, for a total of 18 interviews.

These SSIs and FGDs were conducted in Bangla by the second author. To achieve investigator triangulation [26], the lead author conducted 11 additional SSIs, with the assistance of an experienced local interpreter. All FGDs were conducted inside SoFEA clubs, and SSIs conducted in the nearest private space outside the club. Field notes were taken by KH, UM and a trained research assistant, to document observations regarding the surrounding environment, any non-verbal reactions to questions/discussions, emergent themes and potential follow-up questions to aid later analysis.

As previously described [28], initial questions were generated based on dominant themes from the literature on IYCF and early childcare. Questions in both FGDs and SSIs aimed to gather insight on the following broad topics: (1) early infant care (e.g. popular practices observed in the community, strategies to ensure newborn health); (2) breastfeeding (initiation, duration, exclusivity); complementary feeding (e.g. timing of introduction of complementary foods, food types, food preparation and hygiene); (3) level of community support for IYCF (particularly for young mothers), and (3) perceived support needs. Preliminary Bangla versions of SSI and FGD instruments were pre-tested with a group of SoFEA club members external to the target study population but with similar socio-demographic profiles. Based on pre-testing and consultation with bilingual senior qualitative researchers from BRAC University and BRAC Research & Evaluation Division, questions were then modified to reflect literacy levels, and local linguistic and cultural interpretations. Study tools, including consent materials, underwent several rounds of modification until the research team felt confident that the language was appropriate and relevant for all participants.

While interviews were intended to be open-ended, researchers probed to understand both positive and negative perceptions regarding IYCF and care. For example, participants were asked questions like, “what kind of things help mothers to keep their babies healthy?”; “what makes this difficult?” and “What makes it easy or difficult for mothers to feed foods to babies?” Beginning discussions with hypothetical, depersonalized questions had two advantages. First, the questions could be answered either based on experience (for women who already had children), or more broadly based on observations by adolescents who had not begun childbearing themselves but may help with childcare for younger siblings and/or neighbours. Second, we believed this approach might enable participants to feel more comfortable expressing views and opinions because they were not directly called upon to discuss personal experiences. We recognized the need to remain as neutral as possible while interviewing in order to foster a non-judgemental environment, and to minimize potential impacts of researcher/participant power dynamics shaped by differences in age, education, ethnicity and social status.

While general FGD and SSI scripts were used as a guide, follow-up and probing questions were ultimately driven by the interviewers’ interpretation of participants’ responses, which may have been subject to bias based on underlying assumptions and pre-understandings of the context. To minimize the potential for misinterpretation of participant narratives, KH and UM held extensive debriefing meetings with research assistants at the end of each data collection day to compare notes, discuss emergent findings and to clarify local meanings and cultural/linguistic nuances where necessary. This was a critical step in the research process because while both KH and UM have qualitative research expertise, KH is a non-Bangladeshi and Western-trained researcher whose presence may have influenced participant responses and their willingness to share openly. Working iteratively and collaboratively in a team comprised of both local and foreign researchers was paramount to ensuring high quality and reliable data.

### Data management and analysis

A team of 6 research assistants transcribed all digital SSI and FGD recordings in Bangla, then translated these transcripts into English. As a quality control measure, English transcripts were crosschecked with Bangla recordings and field notes by the second author and two bi-lingual research assistants. FGD and SSI transcripts were coded and analyzed simultaneously using an iterative process guided by the same questions and topics. Data were analyzed in three concurrent stages: data reduction, data display and conclusion drawing/verification [27]. During the data reduction stage, KH and UM reviewed transcripts together to identify broad themes through consensus. We used questions from FGD and SSI scripts as a preliminary guide in a directed content analysis [29]. We further reduced the data by coding transcripts inductively, into sub-themes. During this phase we *coded up*, allowing categories to emerge from the data rather than *coding down*, or forcing pre-determined sub-themes onto the data [30]. To test and verify coding validity, we coded transcripts separately, in English and Bangla. This process not only ensured consistent coding but also drew attention to translation errors and omissions, and differences in cultural interpretations. All data were coded manually, then summarized and displayed in spreadsheets organized by themes and sub-themes. We interpreted data and drew conclusions based on a combination of coding summaries, contextual field notes, and descriptive data provided by direct quotes from participants.

For the purpose of this paper, we chose to focus on isolating the reported challenges to optimal IYCF and caregiving (as opposed to facilitators), as this information shed light on which issues should be prioritized in the development of future nutrition modules. We adopted Bandura’s social cognitive theory (SCT) as a framework to guide data analysis. According to SCT, the reciprocal and bi-directional interactions between people and their environments have a profound impact on health behaviours [31], including infant feeding and caregiving. A key construct of SCT is self-efficacy, the “beliefs in one’s capabilities to organize and execute the courses of action required to produce a given attainment” [32]. Given the programmatic focus on empowerment and capacity building of adolescent girls, this analytical approach was deemed most relevant to future SoFEA programming.

### Ethical considerations

This study was subject to continuing ethical review and was approved by the Social Sciences, Humanities and Education Research Ethics Board at the University of Toronto and by the BRAC University Research Ethics Board.

Upon arrival at each clubhouse, UM described the purpose of the study to SoFEA members as a group. All SoFEA program staff members (both managers and trainers) were asked to leave the premises prior to the consenting process, and for the duration of the FGDs and SSIs, to avoid any undue influence on SoFEA members. Consent protocols were administered verbally, in Bangla; all invitees agreed to participate and provided written or verbal consent. During this process, it was emphasized that program staff would not penalize participants in any way should they refuse participation or decline to answer certain questions and that their SoFEA club membership would not be jeopardized, regardless of the information provided.

## Results

A total of 70 young women participated in one of 29 SSIs or one of 6 FGDs, of whom 44 (63 %) were between 15 and 19 years of age. Of all young women initially approached by SoFEA program staff, only two declined to hear more about the research study. Sociodemographic characteristics of study participants collected at the beginning of each FGD or SSI, following the consent process, are presented in Table 1. At the time of the study, all unmarried participants were living at home and still attending school. While we did not set out to exclude unmarried mothers, there were no young women characterized by this profile in the SoFEA clubs from which we sampled. Among those who were married, none were attending school, and a majority were living in their husband’s familial home, as is customary practice in rural Bangladesh. Among married participants with and without children, about half were unemployed and working as housewives, and half were engaged in income-generating activities such as sewing/tailoring, poultry or cattle rearing, or small business activities.

**Table 1:** Demographic characteristics of participants. Values are presented as median (range)*

Demographics	Median (range), y		
District	Rajshahi	Pabna	Overall
Age at the time of study, by stratum	*N* = 34	*N* = 36	*N* = 70
Unmarried	16.0 (15–23)	17.0 (15–20)	16.5 (15–23)
Married without child	19.0 (15–23)	18.5 (15–20)	19.0 (15–23)
Married with child	22.0 (16–23)	19.0 (18–21)	20.0 (16–23)
Marital age	15.0 (17–21)	14.0 (10–20)	14.0 (10–21)
Age at birth of first child	16.5 (13–21)	15.0 (12–19)	16.0 (12–21)
Years of school education	8.0 (2–16)	7.0 (0–16)	8.0 (0–16)

* Averages and standard deviations are presented elsewhere [22]

Participants in both FGDs and SSIs identified a number of factors believed to influence young women’s ability to properly feed and care for children. There were also a number of perceived support and education needs that emerged from participant narratives; both sets of findings are outlined below. We were unable to characterize any consistent differences in stated perceptions between the three strata of participants, or between the two study sites. To protect participant anonymity, quotes presented in subsequent sections are labelled with participant age group and/or reproductive stratum only.

### Perceived barriers to optimal child feeding and care

Participants linked five major social factors to the provision of optimal feeding and care: early marriage, time allocation conflicts, rural life, short birth spacing, and poverty. Analysis of participant statements suggests that a number of links between social circumstance and self-perceptions operate in these communities. In the text that follows, each theme is described, and presented along with illustrative statements made by participants.

#### Poverty and self-efficacy

There were striking similarities between the expectations and anticipated barriers of unmarried, nulliparous girls, and the experiences described by their married peers with children. Participants associated poverty with difficulties feeding and caring for children because mothers often cannot buy nutritious foods for their children, eat healthy foods themselves (which participants linked to low breast milk supply), send children to school, or provide medicine when children are ill. The anticipated, intrinsic link between poverty and childcare difficulties is described in the following quote:

“We can't do what rich people can. People who are more well off than us, they can take care of children better than us. All mothers do the best they can, but we can’t compare this with what rich people do.”

*- Unmarried participant with no children (>19 y)*

Another participant suggested that despite improved availability of high quality foods in recent years, many families are simply unable to afford them:

“From my view point, more healthy and good quality foods were provided to children in the past. Many good and high quality foods are available in the market now-a-days but not everyone can afford to buy it for their children.”

- *Married participant with no children (<19 y)*

Young mothers’ responses suggest that throughout motherhood, poverty continues to be a major source of frustration, and sometimes guilt, for those who cannot provide adequate foods for their children:

“It feels very bad when the child is seeking something but I am not able to provide him with it. I know these things fill up his stomach and these are good for the child’s health. It feels very bad that we are unable to provide the child with those things.”

*- Married participant with children (<19 y)*

#### Early marriage and autonomy

Some participants entered into arranged marriages as young as 11–12 years of age, at which point they were expected to leave school to live in their husband’s familial home. Marriage was perceived by most participants to be undesirable because childbearing is considered an inevitable next step, and women often have limited autonomy once they are married.

Participants considered early marriage a barrier to optimal child feeding and care because they believed that teenage girls are not prepared physically or emotionally to care for young children. In this context, young women appear to be well aware of the potential psychosocial and economic burden and clinical risks of early marriage and childbearing:

“If a girl gets married at an early age she cannot take care of her husband, then when the baby comes, it causes harm to her body.”

*- Unmarried participant (<19 y)*

A nulliparous married participant echoed this perception, stating “girls [her] age can’t even take care of themselves, so how can they care for a husband and children?”

As a young mother articulates below, while resisting arranged marriages may be an option for some young women, it is more difficult to control the timing of pregnancies once married. One participant described her own experience in a tone of regret:

“We got married at a very young age and gave birth to our children as teenagers. Our bodies have weakened, and we can't take good care of children. We don't even understand what the word "husband" means. That's why young mothers want to make their daughters self-dependent so that they may get some help… if we educate our daughters then they won't have to face these problems. I didn't understand at the time when my parents married me. If I had known more then, I wouldn't have accepted the marriage. I would have continued with my studies.”

*- Married participant with child (FGD)*

#### Rural life and lack of opportunity

Many participants believed that mothers in rural areas face greater difficulties with childcare and feeding than mothers in urban areas. This perception stems from a widely shared perception that in rural areas, mothers are poorer, and therefore experience greater barriers to accessing a variety of foods, resources, and safe, hygienic environments:

“Children in rural areas get sick more than in the urban areas because the environment here is not good… Urban children stay well because they are always neat and clean.”

*- Unmarried participant (FGD)*

Several participants also perceived differences in IYCF practices between rural and urban areas. For example, one unmarried participant stated “since this is a rural area, many mothers feed solid foods at 4–5 months”. Another common perception was that in urban areas, “more women are allowed to go to the markets to buy food” (*married participant*), as opposed to in rural areas, where men typically make most decisions about the household food supply.

#### Time conflicts and women’s work

Both mothers and their nulliparous peers identified time allocation as a barrier to proper feeding and care. Respondents believed this was especially true for women working outside the home, as their work schedule interferes with breastfeeding:

“The working women can’t breastfeed properly. They hire a maid servant for the children and they feed tinned milk.”

- *Unmarried participant (FGD)*

In addition to paid work, study participants highlighted household work as a major consumer of maternal time: “sometimes mothers have too many household jobs so they can’t take care of babies properly” (*married participant with no children, FGD*).

#### Birth spacing and quality of childcare

Participants in all three strata considered short birth spacing to be an obstacle to feeding and care. Participants believed that short birth intervals are problematic for breastfeeding, because “with many young babies there is not enough breast milk to go around” (*married participant with no children, <19 y*). An unmarried participant noted that it is “easier to look after children if there is a bigger gap between two children because the older child can help.”

### Additional challenges specific to breastfeeding

In addition to the perceived barriers outlined above, respondents described several perceptions specific to breastfeeding. For example, participants stated that mothers of higher socioeconomic status tend to feel shy or embarrassed to breastfeed in front of others, and therefore feed tinned milk to babies to maintain social status and to ‘save their prestige’. When discussing the occurrence of breastfeeding in her locality, a married participant with no children (<19 y) remarked, “this is the generation of feeding babies with purchased milk”. Another participant believed that mothers who travel outside of the village for work are more likely to wean their babies onto tinned milk because they come into frequent contact with people of higher social status:

“Many times mothers don’t breastfeed their children (in order) to save their prestige. Sometimes maybe a mother travels to a higher society and there she feels shy to feed the child in front of others. So she feeds the child with tinned milk at that time.”

- *Unmarried participant (FGD)*

Feeding tinned milk was thought to reflect a woman’s social status because financially secure mothers can afford to feed formula, while poor mothers can only afford to breastfeed or feed cow’s milk.

While we did not probe specifically about caesarean deliveries, the topic surfaced in several SSIs and FGDs. Participants reported that undergoing caesarean section is a significant barrier to early initiation of breastfeeding and proper infant care. As one mother remarked, “those who have a caesarean baby, they remain senseless after the child is born”. This young woman recalled her own delivery experience, noting that she was psychologically distressed and in far too much pain to initiate breastfeeding during the first few days after giving birth. For these reasons, she explained, doctors typically prescribe infant formula for newborns following a caesarean delivery.

### Perceived education needs

Participants identified a number of health and nutrition education needs, some related to infant feeding and care, and others related to their own personal health. Unmarried participants were equally interested in these issues as young mothers, in terms of both learning and sharing new knowledge with their peers, as illustrated by this statement from a SSI:

“I want to know about caregiving of children. We are young and we don’t know much about this. This is the time to learn things; I want to learn everything. Those girls who are married and have children, they also want to know about infant care and feeding. What will help to keep the baby healthy and away from diseases? We want to hear about these. It will be helpful for us. We can teach others as well.”

- *Unmarried participant (<19 y)*

Participants were able to be specific in identifying their knowledge needs with respect to infant feeding issues. For example, many expressed a strong desire to learn more about which foods are best for children at specific ages:

“We would like to know about the foods that will keep babies away from diseases so that they will be healthy, can grow up properly, and can study properly. There are many girls who know very little about child nutrition. They give complementary foods before 6 months but shouldn't. They need to know this information.”

- *Married participant with no children (<19 y)*

Participants in all three strata and in both regions expressed the belief that receiving childcare education early is necessary not only because girls become mothers at a young age, but also because many of them play an important role in raising and caring for younger siblings and neighbours. Several married participants with children suggested that it is best to educate girls about childcare during adolescence because girls at this stage of life have the most time available to allocate to learning new things. Several participants explained that once girls are married they are expected to remain in their husband’s familial home and spend most of their time completing household chores. They often do not have the permission or support of husbands and mothers-in-law to take advantage of learning opportunities after marriage. Married girls in particular expressed a need for education about and access to birth control pills and other methods of contraception.

## Discussion

The adolescent girls and young women participating in this study and the research team who analyzed their statements have together identified through the research process a number of key contextual challenges with respect to infant feeding and caregiving. These concerns can be discussed as potentially productive foci for the design of interventions that enhance support for both adolescent mothers and potential mothers-to-be.

Recently there has been a slow but steady increase in the age at which Bangladeshi women first marry [24]; however early marriage is still commonly practiced, particularly in rural regions of the country. Although the legal age for marriage is 18 years for women, early marriage remains common [33]. In the present study, median ages at first marriage and first childbirth were 14 and 16 years, respectively (Table 1). Participant narratives emphasize the need to continue advocacy efforts to delay marriage in Bangladesh and to increase community compliance with existing national laws restricting marriage before the age of 18. Stronger political will and more effective legislative frameworks to ensure enforcement are required to enable broad social change over time. Following marriage, mothers-in-law and male partners often have a strong influence on women’s decision-making regarding timing of marriage and pregnancy [34, 35]. Since early marriage is a historically entrenched practice, and is strongly influenced by family members, there is a need for behaviour-change communication strategies targeted at young married women to engage whole communities in ways that are gender-inclusive across generations.

A perception that rural environments are unclean and unsafe for children likely undermines adolescent girls’ caregiving confidence. It is possible that perceptions of a hostile rural environment may be a motivating factor for young families to move to urban centres on the assumption that urban life will lead to enhanced opportunities for optimal childcare and feeding. Contrary to this perception however, studies have shown that significant socioeconomic and health inequalities exist in both rural and urban Bangladesh [36, 37]. In reality, poor mothers in urban settings are often equally disadvantaged in terms of access to food, services, clean water, and hygienic environments. Many of Dhaka’s urban poor are rural–urban migrants living in crowded, unhygienic informal settlements, and research shows that child mortality is higher among children from migrant families than children born to life-long urban natives [38].

Participants’ dichotomization of rich vs. poor and urban vs. rural appears to impact on perceptions of their own future prospects; many young women believed they will simply be unable to properly feed and care for future children because of these two factors alone. Future interventions should aim to change this perception by teaching adolescent girls how to best utilize the resources available in rural environments. Raising awareness of the challenges faced by their peers living in urban centres could help to deflate romanticized notions of urban life. Further, efforts must also be made to expand and improve access to health services in rural regions so that adolescent girls feel they have adequate childcare resources and support.

Discussions regarding maternal time allocation are important in light of seminal research conducted in the 1980’s, which reported that rural mothers who engage in market activities or other duties incompatible with caregiving tend to decrease the amount of time they allocate to leisure and childcare, which has a negative impact on child nutritional status [39]. A more recent study reported that Nepalese women who worked outside the home were less likely to exclusively breastfeed their infants [40]. Another Nepalese study found that working mothers were significantly less likely to engage in optimal complementary feeding practices [19]. Apart from these studies, the issue of time allocation has received limited attention in recent years. Results of our study point to a need for researchers and programs to re-prioritize maternal time allocation as a key determinant of caregiving behaviours. Efforts to help women re-allocate time to feeding and childcare in this context should involve mothers in-law, as they are usually in charge of delegating household tasks. Another important approach is for programs to target male partners to promote increased male involvement in caregiving and other household responsibilities traditionally performed by mothers.

Previous research in Bangladesh found that younger mothers, and those with higher parity, breastfeed for shorter durations than their peers [41]. These findings, combined with statements by participants in our study, suggest a need for increased access to family planning services for young women. While contraception use by adolescent girls is increasing in Bangladesh, access to contraception and family planning education remains an unmet need across the country [42]. In other low-income settings, linkage of messages on family planning and child survival has the potential to motivate and educate more effectively [43], and this may also be true in Bangladesh. Again, since husbands and mothers-in-law are often key family planning ‘gatekeepers’, wide community engagement is crucial for building an environment for healthy family planning.

Empirical studies in Bangladesh and elsewhere have documented negative associations between poverty and infant feeding practices [44–47]. Other qualitative research with low-income mothers has found financial constraints to be a perceived barrier to optimal feeding practices [48], however we are not aware of any published studies that considered the perceptions of adolescent girls and young women, specifically. Recent studies of food insecurity, an indicator and direct consequence of poverty in many low-income groups, suggest that it can have major influence on adolescent perceptions [49] and contributes to maternal stress and directly undermines self-efficacy and infant feeding [50, 51].

Study findings indicate that self-efficacy may be a critical unmeasured factor linking several dimensions of participants’ social context with negative self-perceptions and low expectations of motherhood, feeding and childcare. The presence of persistent life stressors, including poverty, is known to place women at considerably high risk for low maternal self-efficacy [32]. Low maternal self-efficacy is associated with poor infant feeding outcomes [52, 53], can disrupt mother-infant bonding [54] and has been linked to anxiety, depression, and demotivation [55]. Maternal self-efficacy has also been associated with women’s ability to provide a stimulating and nurturing child-rearing environment [56].

Although this study did not score individuals on self-efficacy, the findings are consistent with the possibility that perceived maternal inadequacy begins to develop prior to motherhood, at least with respect to care and feeding. The indications that, prior to childbearing, many girls and young women anticipate that they will be inferior caregivers due to poverty are of critical concern because behaviour change and positive practices are founded on self-efficacy and agency. Taken together, findings suggest that further research to understand the processes through which girl-child and adolescent self-efficacy is enhanced or undermined has the potential to inform maternal, infant and child health interventions in this setting. They also highlight an urgent need for future IYCF and care interventions to target nulliparous adolescent girls.

### Limitations of the study

This study had several limitations. First, we intentionally recruited study participants through an existing community-based program in order to gain programmatically relevant information, however it is possible that program exposure effects on the findings may render them non-generalizable to urban or other areas where such programs are absent. For example, the reported perception that early marriage impedes proper feeding and caregiving may be an artefact of the SoFEA program and its emphasis on the benefits of delaying marriage. This poses an interesting research question for future studies: are the girls and young women highlighting early marriage as a barrier to caregiving because early marriage is discouraged by SoFEA program staff, or did the girls envision early marriage as problematic prior to their involvement in the SoFEA clubs?

Second, given that program staff members were involved in the recruitment process, the sample of girls invited to participate may have been biased, as those most likely to discuss positive aspects of the SoFEA program may have been favoured for selection. However, because this was not a formal program evaluation and there were no nutrition or childcare education components in the program at the time, any pressure to showcase the SoFEA program in a positive light was likely minimal.

Third, we have made inferences about young women’s self-efficacy with respect to feeding and caregiving because the concept emerged through our specific methods of qualitative data collection and analysis. However, since we did not set out to measure self-efficacy in any direct, systematic way this interpretation should be validated with such measures.

Lastly, it is possible that different results might emerge if we had conducted a greater number of FGDs with a larger sample of participants.

## Conclusions

Taken together, the themes expressed by the participants in this study echo the findings of previous research in Bangladesh [35] that poverty and vulnerability to economic crisis are significant barriers to later marriage and delayed childbearing, even among girls and young women aware of the risks associated with early marriage and childbearing. Given the apparent links between perceived poverty, early marriage, unmet need for family planning, and low caregiving self-efficacy, future interventions to improve maternal, neonatal and child outcomes will likely be most successful if combined with efforts to empower girls financially. Education and support offered through such integrated interventions could enhance the confidence of young girls and thus the quality of their caregiving practices when they eventually do become mothers. As we have reported previously [23], simple delivery of public health messages on infant care and feeding without any other form of support or counselling is not sufficient, as some adolescent girls may not understand the intended meaning of such messages. Programs might also consider utilizing a peer-to-peer model, in which older or more experienced young women are trained as peer leaders who provide support for reproductive health, infant feeding and caregiving. These individuals would be best positioned to empathize with the needs and experiences of other young women in their communities and serve as effective positive role models.

The evidence presented here suggests that integration of infant care and nutrition modules into existing adolescent empowerment programs would be beneficial both for adolescent mothers and girls who have not yet had children. However, given that the primary goal of BRAC’s SoFEA program is to promote delayed marriage and childbearing, recommendations for this integration may seem counter-intuitive: should such programs target adolescent girls with training to help them become better caregivers, or should efforts be put exclusively towards increasing the age at first marriage? While recognizing this tension in setting appropriate program goals and priorities, we argue that the two approaches do not have to be mutually exclusive and should be integrated to better fit the lived experience and multiple expressed needs of adolescent girls in this context. Although empowering adolescents to delay marriage should bring benefits for future generations of girls, immediate efforts to support young mothers will have potential value in the meantime. A need to improve the care of infants born to young girls is one of many immediate public health challenges that arise from the present reality that many girls continue to be married and become mothers at very young ages. Provision during adolescence of appropriate education, services and financial aid to support best practices for infant feeding and childcare could significantly improve the health and nutrition security of young children in rural Bangladesh.

## Footnotes

1 formerly known as Bangladesh Rural Advancement Committee
